# Exercise benefits yourself and your offspring: a mini-review

**DOI:** 10.3389/fcell.2025.1606790

**Published:** 2025-05-30

**Authors:** Kun Wang, Jiajia Zhao, Yanqiu Wang, Mairu Liu

**Affiliations:** ^1^ Faculty of Physical Education, China West Normal University, Nanchong, China; ^2^ School of Physical Education, Central China Normal University, Wuhan, China

**Keywords:** maternal exercise, offspring, metabolism, neuron development, immunity

## Abstract

Regular physical activity is widely recognized for its systemic health benefits, extending beyond physical fitness to influence metabolism, immunity, and neurophysiology. Pregnancy is a physiologically unique period characterized by dynamic immunometabolic changes that are crucial for maternal and fetal health. Maternal exercise during this window offers a non-pharmacological strategy to enhance maternal wellbeing and optimize offspring development. This review summarizes recent advances in understanding the effects of maternal exercise on both pregnant women and their offspring. In mothers, exercise improves metabolic profiles, modulates inflammatory responses, supports neuroplasticity, and promotes skeletal health. In offspring, maternal exercise confers long-term benefits including improved glucose metabolism, enhanced neurogenesis, cognitive development, and immune resilience. Mechanistically, these effects are mediated through molecular pathways such as placental superoxide dismutase 3 (SOD3) upregulation, adenosine 5′-monophosphate-activated protein kinase/ten-eleven translocation (AMPK/TET) signaling in the fetal liver, and exercise-induced circulating factors like Apelin and SERPINA3C, which contribute to epigenetic remodeling and tissue-specific programming. Despite growing evidence, gaps remain in understanding the optimal intensity, timing, and molecular mediators of maternal exercise, particularly regarding long-term immune and neurodevelopmental outcomes in offspring. Future studies leveraging multi-omics approaches are needed to elucidate cross-organ signaling mechanisms and identify therapeutic targets to mimic exercise-induced benefits. Overall, maternal exercise emerges as a safe, accessible intervention with significant potential to improve maternal-fetal health and reduce offspring disease risk across the lifespan.

## 1 Introduction

Regular exercise is a health-promoting lifestyle generally recommended to reduce the risk of various disorders. Growing evidence shows the multiple benefits of exercise, which extend beyond physical fitness and can exert positive effects on the metabolism, immunity, and nervous system (Kusuyama et al., 2020; Hayman et al., 2023; Davenport et al., 2018). However, the deeper underlying mechanism of exercise-induced effects remains unclear, hindering the development of alternative drugs that can reproduce the exercise-induced effects.

The prenatal period encompasses a critical window for the future healthy development of offspring (the Barker Hypothesis). Thus, investigating the effects of maternal exercise during pregnancy on offspring throughout intrauterine and postnatal development is also an interesting topic (Muglia et al., 2022). Emerging evidence supports that moderate exercise by mothers during pregnancy benefits their children. The US Department of Health and Human Services recommends that pregnant women insist on a minimum of 150 min per week of moderate-intensity exercise (Piercy et al., 2018). However, only a minority of pregnant women meet the recommendations (Hayman et al., 2023; Davenport et al., 2018).

In this review, we firstly introduced the benefits of exercise on pregnant women focusing in their common body conditions, which include recent findings of exercise-induced effects on body metabolism, neuron system, immune system, and skeletal system. We also review recent studies of maternal exercise-induced effects on offspring. We also discuss the present challenges and future directions for studying exercise.

## 2 Benefits of exercise on mothers

**FIGURE 1:**
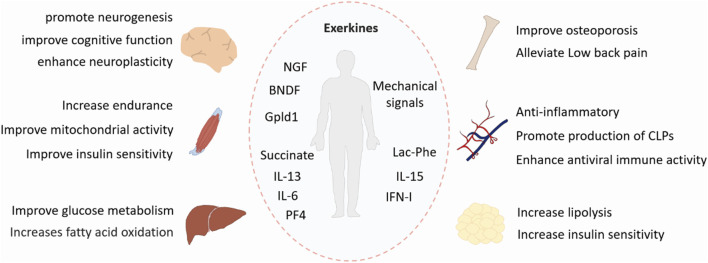
Benefits of exercise. Exercise has systemic positive effects on multiple tissues, including the skeletal system, muscle, adipose tissue, liver, and brain. Exerkines are exercise-induced factors that exert their effects through endocrine, paracrine, or autocrine pathways. BDNF, brain-derived neurotrophic factor; NGF, nerve growth factor; Gpld1, glycosylphosphatidylinositol-specific phospholipase D1; IL-6, interleukin-6; IL-13, interleunkin-13; IL-15, interleukin-15; IFN-I, type I interferon; Lac-Phe, *N*-lactoyl-phenylalanine; PF4, platelet factor 4; CLPs, common lymphoid progenitors.

Physical exercise is a well-known non-pharmacological treatment to improve various disorders. It produces systemic health benefits by affecting multiple tissues, including the skeletal system, muscle, adipose tissue, liver and brain (Figure 1). Therefore, these exercise-induced effects and the underlying mechanisms must be studied from a holistic perspective.

### 2.1 Improve body metabolism

The global incidence of obesity and diabetes has risen sharply, and exercise is a key non-pharmacological intervention to improve metabolic health. Traditionally, the benefits of exercise have been attributed to skeletal muscle (Egan and Zierath, 2013; Jonathon et al., 2023), which releases myokines and metabolites during activity (Martin et al., 2023). For example, succinate (Reddy et al., 2020) and IL-13 (Nelson et al., 2020) are secreted by muscle during exercise and contribute to enhanced glucose tolerance, mitochondrial activity, and endurance.

However, recent studies highlight that other tissues also mediate exercise benefits (Stanford et al., 2015a). Adipose tissue responds to exercise in a time-of-day dependent manner, as shown by Pendergrast et al. (2023), with fat mobilization occurring only during nocturnal activity in mice. Exercise also modulates cardiac metabolism by reducing glycolytic activity (Gibb et al., 2017) and reshapes the gut microbiome (Jonathan et al., 2024), thereby improving endurance. Furthermore, Li et al. identified the metabolite N-lactoyl-phenylalanine (Li et al., 2022), which suppresses appetite and reduces obesity, though its cellular origin remains unclear. A meta-analysis also suggests that combining exercise with metformin enhances glucose regulation in diabetic patients (Zhao et al., 2024).

In humans, maternal exercise during pregnancy lowers gestational weight, reduces cesarean risk (The International Weight Management in Pregnancy (i-WIP) Collaborative Group, 2017; Wang et al., 2017), and decreases the incidence of gestational diabetes mellitus (GDM) (Wang et al., 2017). It also induces long-term liver mitochondrial adaptations in GDM mothers, potentially delaying metabolic complications later in life (Stevanović-Silva et al., 2021).

Advancements in multi-omics technologies have accelerated this field. Sato et al. mapped the exercise-induced metabolome across tissues and time points, while the Molecular Transducers of Physical Activity Consortium developed a comprehensive database spanning transcriptomic to epigenomic changes across multiple tissues during endurance training (Sato et al., 2022). These resources offer powerful tools for deciphering the complex molecular responses to exercise and identifying potential therapeutic targets (MoTrPAC Study GroupLead AnalystsMoTrPAC Study Group, 2024).

### 2.2 Improve nervous system

Exercise exerts profound benefits on the nervous system, influencing both the central and peripheral components. A large body of evidence shows that physical activity promotes neurogenesis, particularly in the hippocampus (Liu et al., 2019). Van Praag et al. demonstrated that running enhances dentate gyrus neurogenesis in mice, improving memory and learning performance (van Praag et al., 1999). Aerobic exercise has been shown to most effectively stimulate adult hippocampal neurogenesis (Nokia et al., 2016), which is also essential for maintaining cognitive function in aging (Zhou et al., 2021). Exercise also enhances neuroplasticity (Yamaguchi et al., 2016), partly by upregulating neurotrophic factors. Notably, exercise increases brain-derived neurotrophic factor (BDNF) expression (Sleiman et al., 2016; Adlard et al., 2005), which supports synapse formation, plasticity, and cognitive enhancement (Hempstead, 2015; Casarotto et al., 2021; Fang et al., 2003; Anastasia et al., 2013; Kowianski et al., 2018). Additionally, nerve growth factor (NGF) activated by exercise binds to TrkA receptors, promoting neuronal survival and synaptic modulation (Chao et al., 2006; Saragovi et al., 1998; Hall et al., 2018). Interestingly, exercise not only acts as a metabolic challenge but also initiates brain-driven metabolic regulation (Hwang et al., 2023; Gautron et al., 2015). For instance, BDNF influences systemic metabolism (Fulgenzi et al., 2020; Xu and Xie, 2016), and exercise stimulates hypothalamic POMC neurons (Kang et al., 2021), leading to thermogenesis via adipose tissue mitochondrial activation. Exercise improves cognitive functions, including memory, learning, and decision-making (Augusto-Oliveira et al., 2023). Horowitz et al. found that plasma from exercise-trained aged mice improves cognition and neurogenesis in sedentary peers, with Gpld1 identified as a key circulating factor (Horowitz et al., 2020). Similarly, platelet factor 4, higher in younger individuals, reduces neuroinflammation and enhances cognition in aged mice (Schroer et al., 2023). Moreover, exercise mitigates neurodegenerative conditions (Zhang et al., 2019). Long-term physical activity alleviates cognitive impairment in Alzheimer’s disease mice by enhancing lysosomal function and promoting amyloid-beta clearance (Wang et al., 2022). Mechanistically, exercise facilitates nuclear translocation of TFEB, increases interaction with AMPK-mediated acetyl-CoA synthetase 2, and boosts lysosomal gene transcription.

In summary, exercise promotes neuronal development, synaptic plasticity, metabolic regulation, and cognitive resilience, highlighting its therapeutic potential for neurodevelopmental and neurodegenerative conditions.

### 2.3 Improve immunity

The immune system plays essential roles in defense, regulation, and homeostasis, and exercise has emerged as a powerful modulator of immune function (Friedrich, 2008; Watts, 2012). One of the most consistent findings is that regular physical activity helps reduce systemic inflammation (Gleeson et al., 2011), which is particularly beneficial in chronic metabolic disorders such as type 2 diabetes (Papagianni et al., 2023). This anti-inflammatory effect is supported by evidence showing that exercise downregulates pro-inflammatory signaling pathways and enhances anti-inflammatory immune responses, partly through epigenetic and metabolic modulation of immune cells (Nini et al., 2024). Aging-related increases in inflammatory activity can also be attenuated by exercise, highlighting its role in immune rejuvenation (Ling et al., 2023).

Beyond controlling inflammation, exercise promotes immune cell production and activity (Shen et al., 2021). Mechanical stimulation during physical activity can trigger bone marrow niche cells to release factors that support lymphoid progenitor expansion (Tengfei et al., 2024). Additionally, exercise enhances innate antiviral responses, such as increased type I interferon production, and promotes the expansion of regulatory T cells in muscle tissue (Langston et al., 2023). These cells help maintain immune balance and support tissue integrity by preventing excessive inflammatory responses that could lead to cellular damage.

Given these immunomodulatory properties, exercise is increasingly recognized as a valuable adjunctive therapy in cancer (Fiuza-Luces et al., 2024; Kathryn et al., 2019). It has been shown to improve quality of life (Anouk et al., 2024), physical function (Scott et al., 2018), and immune competence (Kurz et al., 2022) in cancer patients. Exercise can lower recurrence risk (Soldato et al., 2024), enhance tumor immune surveillance, and improve treatment outcomes. Mechanistically, this involves the release of cytokines such as IL-15 (Kurz et al., 2022), which supports T cell mobilization and tumor infiltration, strengthening anti-tumor immunity.

Importantly, exercise also contributes to long-term health and longevity, regardless of disease status, by promoting systemic immune balance. Together, these findings support the role of regular physical activity as a low-cost, non-pharmacological strategy to enhance immune defense, reduce chronic inflammation, and support disease prevention and recovery, particularly in aging and cancer contexts (Jessica et al., 2024; Lavery et al., 2023).

### 2.4 Effects on skeletal system disorders

The skeletal system is fundamental for movement and structural support, and exercise plays a critical role in its development, maintenance, and rehabilitation (Lee et al., 2014; Huiskes et al., 2000). Physical activity has long been recommended for managing skeletal disorders such as osteoporosis and low back pain (LBP) (Kise et al., 2016; Pagnotti et al., 2019; Breda et al., 2021; Owen et al., 2020).

In osteoporosis, particularly among postmenopausal women (Pagnotti et al., 2019; Courteix et al., 1998), various exercise modalities have shown beneficial effects on bone mineral density (BMD) (Mohammad Rahimi et al., 2020). Resistance and impact training are especially effective in improving bone strength and functional performance (Watson et al., 2018), while mind–body exercises (Zhang et al., 2021), such as Tai Chi, have been associated with BMD improvements in the lumbar spine and femoral neck, particularly with long-term practice (Chow et al., 2018; Sun et al., 2016). Mechanistically, bones respond to mechanical loading (Huiskes et al., 2000), where osteoblasts sense strain through mechanosensitive ion channels like PIEZO1/2 (Sun et al., 2019; Wang et al., 2020). In addition to mechanical signaling, moderate exercise has been shown to influence bone formation through sympathetic cholinergic nerve fibers (Gadomski et al., 2022) and epigenetic modifications (Chen et al., 2021), offering insights into how physical activity promotes skeletal adaptation at a molecular level.

For LBP, a condition increasingly prevalent and economically burdensome, intervertebral disc (IVD) degeneration is often a primary cause. Exercise has emerged as a non-invasive strategy for promoting IVD regeneration (Sasaki et al., 2012). Experimental models show that exercise stimulates the proliferation of IVD progenitor cells and increases glycosaminoglycan content (Ueta et al., 2018), enhancing disc hydration and matrix integrity. In humans, early-stage physical activity yields modest but significant improvements in disability related to recent-onset LBP (Fritz et al., 2015). Specific movement therapies, such as motor control exercise (Saragiotto et al., 2016) and moderate intensity aerobic training (Belavý et al., 2017), have shown low to moderate efficacy in reducing chronic LBP symptoms and improving long-term disc function (van Dillen et al., 2021), especially in individuals with less physically demanding occupations (Hayden et al., 2020).

Overall, exercise serves as a mechanically and biologically active intervention for skeletal health, benefiting both bone density and spinal disc integrity, and offers a promising alternative or adjunct to pharmacological and surgical treatments for skeletal disorders.

## 3 Benefits of maternal exercise on offspring

**FIGURE 2:**
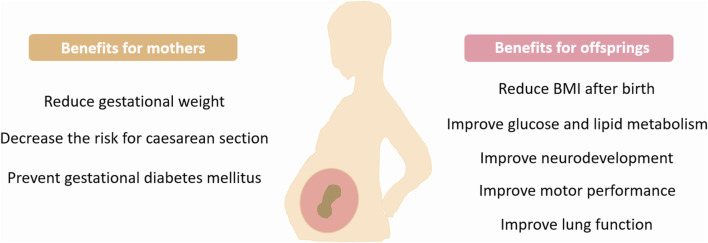
Benefits of maternal exercise. Exercise during pregnancy provides many health benefits for mothers and children. BMI, Body mass index.

Maternal exercise during pregnancy exerts multiple beneficial effects on offspring and confers protection against the development of various disorders. However, more studies are required to reveal the maternal-exercise-induced long-term effects on offspring (Figure 2).

### 3.1 Improved metabolic health in offspring

Increasing evidence suggests that an adverse intrauterine environment is strongly associated with a higher risk of obesity and diabetes in offspring (Kusuyama et al., 2020; Sales et al., 2017). In contrast, maternal exercise has emerged as a promising non-pharmacological intervention to improve offspring metabolic health (Harris et al., 2018).

In humans, maternal physical activity during pregnancy is associated with favorable postnatal outcomes, including reduced offspring subcutaneous fat mass (Clapp, 1996), lower BMI (Mourtakos et al., 2015; Jevtovic et al., 2005), and improved body weight regulation into early childhood. Importantly, maternal exercise has been shown to influence the metabolic function of offspring mesenchymal stem cells (MSCs) (Jevtovic et al., 2024), enhancing glucose and lipid metabolism (Chaves et al., 2022), with resistance training showing the most prominent effects (Jevtovic et al., 2023).

Animal studies further confirm that maternal exercise mitigates the adverse metabolic effects of a maternal high-fat diet (Stanford et al., 2017), improving glucose tolerance and liver metabolism in offspring (Zhang et al., 2023). However, the timing of exercise is critical; benefits are most evident when exercise is performed both before and during gestation, but not if limited to either period alone (Stanford et al., 2015b; Sheldon et al., 2016).

At the molecular level, recent research has identified several key pathways through which maternal exercise benefits fetal development. These include the vitamin D receptor-mediated increase in placental superoxide dismutase 3 (SOD3) (Kusuyama et al., 2021), which activates adenosine 5′-monophosphate-activated protein kinase/ten-eleven translocation (AMPK/TET) signaling and promotes DNA demethylation of glucose metabolism genes in fetal liver (Bae-Gartz et al., 2020). Additionally, exercise-induced circulating factors such as Apelin (Jun Seok et al., 2020) and SERPINA3C (Li et al., 2025) play crucial roles in enhancing brown adipose tissue development and reducing inflammation via PI3K-TET1-Klf4 signaling in fetal adipose tissue (Li et al., 2025).

Together, these findings highlight maternal exercise as a powerful modulator of epigenetic programming and cellular metabolism in offspring, offering long-term protection against metabolic disorders.

### 3.2 Promotion of neuron development in offspring

Maternal exercise during pregnancy has been increasingly recognized to promote not only maternal neurogenesis but also enhance neurodevelopmental outcomes in offspring, particularly in cognitive, behavioral, and motor domains (Wiebe et al., 2015; Labonte-Lemoyne et al., 2017). Studies report that offspring of physically active mothers show more mature neonatal EEG patterns (Labonte-Lemoyne et al., 2017), reduced neural immaturity markers (Clapp et al., 1999), and higher cognitive performance, including elevated IQ levels during infancy (Domingues et al., 2014).

The neuroprotective and neuroenhancement effects of gestational physical activity are likely mediated by multiple mechanisms. Exercise improves fetal cerebral oxygenation (Moreno-Fernandez et al., 2020), promotes synaptogenesis (Yau et al., 2019), and enhances hippocampal neurogenesis, leading to long-term benefits in learning-memory capability (Ayfer et al., 2012), and emotional regulation (Kim et al., 2024). Some findings indicate sex-specific effects, with male and female offspring showing distinct cognitive and neural responses (Yau et al., 2019). Additionally, pre-pregnancy exercise may confer resilience against prenatal stress (Nakahara et al., 2021) and reduce neurodevelopmental issues (Klein et al., 2019) such as sleep or behavioral disturbances (Nakahara et al., 2021).

On a molecular level, maternal exercise has been shown to suppress neurotoxic markers like tau phosphorylation and oxidative stress (Klein et al., 2020), while increasing neurotrophic factors such as BDNF and mature neurotrophic proteins (Park et al., 2021), which contribute to enhanced neurogenesis and synaptic plasticity in the offspring brain (Akhavan et al., 2011). Amyloid precursor proteins (Mohammad et al., 2024) and hippocampal plasticity pathways have also been implicated in mediating these effects.

Motor development benefits have also been reported, with offspring demonstrating improved neuromotor performance in infancy (McMillan et al., 2019) and even into later childhood (Ferrari et al., 2023). These motor improvements may be linked to increased maternal BDNF levels during late pregnancy, which can cross the placenta and influence fetal brain development. However, some findings remain inconsistent, with certain long-term studies reporting no significant differences in motor outcomes (Ellingsen et al., 2020).

Overall, prenatal exercise is a promising, low-risk intervention that supports neural development and functional maturation in offspring. Despite encouraging findings, mechanistic understanding remains limited, highlighting the need for further research into how maternal physical activity programs neurodevelopmental trajectories (Na et al., 2022; Zhou et al., 2022).

### 3.3 Immunomodulation in offspring

Pregnancy is characterized by trimester-specific immunometabolic adaptations, essential for maintaining maternofetal homeostasis and supporting healthy gestation. These physiological changes include dynamic modulation of inflammatory responses (Mor et al., 2011; Kalagiri et al., 2016), which may be influenced by maternal lifestyle factors such as physical activity. Given the role of exercise-induced cytokines (exerkines) in systemic immunoregulation, maternal exercise could serve as a potential non-pharmacological strategy to modulate inflammation during pregnancy. Exerkines refer to cytokines, peptides, and proteins induced by exercise, which exert their effects throughout the body through blood circulation, regulating various physiological and metabolic processes (Sabaratnam et al., 2022).

Emerging, though limited, evidence suggests that maternal exercise reduces systemic inflammation in pregnant women (Wang et al., 2015; Hawkins et al., 2014). Light to moderate physical activity has been associated with lower levels of C-reactive protein (CRP) (Tinius et al., 2017) and pro-inflammatory cytokines such as interleukin-6 (IL-6) and tumor necrosis factor-alpha (TNF-α) (Acosta-Manzano et al., 2019). However, findings on vigorous exercise remain inconsistent, with some data indicating elevated IL-6 or IL-1β (Acosta-Manzano et al., 2020), emphasizing the importance of exercise intensity and the need for cautious interpretation due to statistical variability. Some studies suggest that moderate-intensity exercise offers the most favorable inflammatory profile, balancing immune activation and suppression (Dhar et al., 2024).

Animal studies further support these findings. Prenatal exercise in rodent models has been shown to increase BDNF and decrease inflammatory markers in offspring exposed to brain injury, suggesting that maternal physical activity confers neuroprotection through enhanced antioxidant and anti-inflammatory pathways (Gorgij et al., 2021).

Despite these promising observations, research on immunomodulation by maternal exercise remains limited, particularly regarding long-term effects on offspring immune and neurodevelopmental health. Future studies are needed to clarify the dose-response relationship between exercise intensity and immune outcomes, and to elucidate the role of maternal exerkines in mediating maternal-fetal immune communication (Acosta-Manzano et al., 2020; Adamo et al., 2024).

### 3.4 Other benefits of maternal exercise to offspring

Beyond the improvements in metabolic health and neuronal function, maternal exercise has many other beneficial effects on offspring, such as improving hypertensive disorders of pregnancy (Barakat et al., 2016), reducing the risk of cesarean section (Di Mascio et al., 2016; Owe et al., 2016) and heart protection (Reihaneh et al., 2023). Although research in these aspects is not as extensive as that on the effects of maternal exercise on offspring metabolism and neurodevelopment, several randomized clinical trials have reported relevant findings. Musakka et al. (2024) reported as well that maternal exercise during pregnancy, when practiced three or more times per week, is associated with a reduced risk of asthma in offspring. Carlsen et al. (Gudmundsdóttir et al., 2022) suggested that physical activity in the first half of pregnancy is linked to increased lung function in the child. Moreover, Owe et al. (2016) found that regular exercise during pregnancy is associated with reduced risk of acute cesarean section for mothers. Additionally, the meta-analysis from Davenport et al. (2018) indicates that maternal exercise is not associated with adverse childhood complications, but it is associated with reduced odds of macrosomia. Macrosomia refers to infants with a birth weight exceeding 4,000 g, and it is associated with several maternal and fetal complications such as maternal birth canal trauma, shoulder dystocia, and perinatal asphyxia (Araujo Junior et al., 2017; Nguyen and Ouzounian, 2021). Zhang et al. also reported that maternal exercise can alleviate oxidative stress and the impairment of endothelium-dependent vasodilatation, thereby improving vascular function in hypertensive offspring.

Although current findings tentatively indicate various potential enhancing effects of maternal exercise during pregnancy, yet conclusions are constrained by methodological limitations including small sample sizes and inconsistent assessment protocols. Moreover, research examining the impacts of prenatal exercise on offspring across different offspring age groups remains limited, with underlying mechanisms poorly understood. Therefore, addressing these research gaps holds significant clinical value for establishing evidence-based guidelines for prenatal health management.

## 4 Risks of maternal exercise during pregnancy

While prior research has established the benefits of maternal exercise for offspring, its potential association with miscarriage risk warrants clarification. Our comprehensive literature review found no evidence that exercise during pregnancy increases miscarriage risk. However, the lack of documented evidence does not preclude this possibility. Previous research indicated that high-intensity exercise can negatively affect placental blood flow (Salvesen et al., 2012). While some studies suggest strenuous exercise could be safe for pregnant women, but only for athletes who are well trained before pregnancy (Titova et al., 2024). Moreover, vigorous leisure activity is associated with reduced birth weight, suggesting a cautious engagement in vigorous exercise during pregnancy (Leet and Flick, 2003; Evenson et al., 2014; Mottola et al., 2018). The Australian guidelines proposed by Brown et al. (2022), aligned with recently published international standards and professional recommendations, outline contraindications and warning signs for prenatal and postnatal physical activity/exercise. Pregnant individuals should undergo individualized risk assessments and prioritize moderate-to-low intensity exercise while monitoring for pregnancy-related complications (Vargas-Terrones et al., 2019; Bull et al., 2020). Absolute contraindications may include: Poorly controlled metabolic disorders (Type 1 diabetes or Thyroid disease); Cardiovascular/Respiratory disorders; Pre-eclampsia; Cervical insufficiency or ruptured membranes; Persistent second or third trimester bleeding; Placenta previa; Intrauterine growth restriction; Multiple gestation (triplets or higher number). Overall, further research is warranted to systematically evaluate exercise-related risks during pregnancy through comprehensive risk stratification and establish standardized risk assessment protocols for prenatal exercise to prevent adverse pregnancy outcomes while optimizing maternal-fetal health outcomes.

## 5 Conclusion

Regular exercise can improve whole-body health, but the systemic effects and the underlying molecular mechanisms remain incompletely understood. Various sequencing methods that have emerged in recent years can help us further understand the exercise-induced systemic effects. In fact, scientists around the world have provided multiple databases for studying the exercise-induced cross-organ effects under different conditions. These datasets serve as valuable resources for understanding the multi-tissue molecular effects of exercise. However, current studies do not consider the effects of exercise on pregnant mammals and their offspring. Future study may profile the multi-omic sequence across tissues of pregnant mammals with or without exercise, which must be helpful for exploring the molecular effects of exercise on pregnant mothers and their offspring.

Additionally, despite the multiple benefits of exercise introduced here, an active lifestyle, as well as insistence on exercise, may be difficult for most individuals because of busy work or owing to age, disease, or other reasons. Therefore, one of the ultimate goals of sports medicine research is to identify the key regulators and factors (e.g., peptides, metabolites, and cytokines) that are induced after exercise. They may be developed as potential therapeutic agents to mimic beneficial effects in the absence of physical training. Moreover, the multi-omics profiles for exercise under any of the conditions mentioned above may be pivotal for identifying the promising target.
